# Cannabinoid Receptor 1 Blockade Attenuates Obesity and Adipose Tissue Type 1 Inflammation Through miR-30e-5p Regulation of Delta-Like-4 in Macrophages and Consequently Downregulation of Th1 Cells

**DOI:** 10.3389/fimmu.2019.01049

**Published:** 2019-05-10

**Authors:** Kathryn Miranda, Pegah Mehrpouya-Bahrami, Prakash S. Nagarkatti, Mitzi Nagarkatti

**Affiliations:** Department of Pathology, Microbiology and Immunology, School of Medicine, University of South Carolina, Columbia, SC, United States

**Keywords:** CB1, DLL4, macrophage, miR-30e-5p, Notch, obesity, Th1

## Abstract

Obesity is characterized by chronic low-grade inflammation that contributes to development of cardiometabolic disorders. Cannabinoid receptor 1 (CB1) antagonists attenuate diet-induced obesity (DIO) and related inflammation, although the precise anti-inflammatory mechanisms involved have not been fully explored. In the current study we used a mouse model of DIO intervention to determine the microRNA (miRNA, miR)-mediated anti-obesity and anti-inflammatory effects of the CB1 antagonist, AM251. DIO mice that were fed high-fat diet (HFD) for 12 weeks were treated with AM251 (10 mg/kg) for an additional 4 weeks. HFD + AM251 mice experienced rapid and prolonged weight loss and reduced inflammatory M1 adipose tissue macrophage (ATM) infiltration. To investigate miRNA-mediated regulation of ATMs, F4/80+ cells from stromal vascular fractions (SVF) of epididymal fat were subjected to miR microarray analysis. Several miRs were differentially expressed in AM251-treated mice that were independent of calorie restriction. Prominently, miR-30e-5p was upregulated in ATMs from HFD + AM251 mice while the miR-30e-5p target, DLL4, was downregulated. Consistent with a decrease in DLL4-Notch signaling, fat storage and pro-inflammatory cytokine/chemokine expression was reduced following AM251 treatment. Furthermore, we found that AM251-treated macrophages can suppress DLL4-mediated Th1 polarization in CD4+ T cells. Together these data demonstrate that blocking CB1 receptors leads to upregulation of miR-30e-5p and down regulation of DLL4 in ATMs, which in turn suppress DLL4-Notch signaling-induced polarization of inflammatory Th1 cells and adipocyte energy storage. This combined effect of ATMs and T cells leads to an anti-inflammatory state and attenuation of DIO. These data support therapeutic potential of miR-30 in the treatment of cardiometabolic disorders.

## Introduction

Obesity is a chief contributor to the global health burden as the World Health Organization estimates nearly 40 % of the population is overweight or obese. Obesity presents a risk to development of comorbid disorders such as type II diabetes, cardiovascular disease, and cancer (1, 2). Causes of obesity can be environmental—such as diet composition, overconsumption, and physical inactivity; or genetic—such as having a genetic mutation in the genes encoding the satiety hormone leptin or its receptor (1, 3). All things considered, environmental factors involving poor diets, such as high-fat diets (HFDs), are a driving force in the ongoing expansion of the disease.

Therapeutic strategies employed to combat diet-induced obesity (DIO) include lifestyle modifications and pharmacological interventions. While lifestyle changes would be most effective to curtail the obesity pandemic, such changes can be difficult to attain and/or maintain and combinations with pharmacological therapy have been shown to improve health outcomes (4). Notably, the endocannabinoid (eCB) system is a major target for pharmacological therapy as its over activation is associated with obesity. The eCB system is comprised of cannabinoid receptors type 1 and 2 (CB1 and CB2) and their endogenous ligands called “endocannabinoids.” CB1 expression is highly concentrated in the central nervous system and to a lesser extent in peripheral metabolic tissues, as well as, immune cells. Thus, CB1 is highly involved in appetite and metabolic regulation. Nearly two decades ago, it was discovered that activation of CB1 increases appetite and could promote development of obesity (5). Thus, CB1 knockout mice were found to be resistant to development of DIO [(6), p. 1]. This led to the use of SR141716A (aka Rimonabant), a CB1 antagonist that reduces food intake, to treat DIO [(7, 8) p. 1; (9) p. 1]. Unfortunately, adverse neuropsychiatric side effects caused this drug to fail to gain US Food and Drug Administration approval (10). However, research into unveiling the mechanisms by which CB1 blockade attenuates obesity has continued in the hopes of identifying additional target pathways for obesity treatment.

Another opportunity for attenuating obesity is targeting inflammation. Obesity is well-characterized as having a state of chronic low-grade inflammation (11). Increased adiposity initiates a switch from anti-inflammatory pro-insulin sensitive type 2 immune surveillance, to pro-inflammatory type 1 metabolic inflammation, which promotes metabolic dysfunction (11). In adipose tissue, excess fat storage and adipocyte death leads to recruitment, polarization, and proliferation of pro-inflammatory M1 adipose tissue macrophages (ATMs), which secrete cytokines such as TNFα, IL-6, CCL2, and IL-1β that decrease adipocyte insulin sensitivity and promote tissue damage (12, 13). Additionally, M1 ATMs can act as antigen presenting cells to stimulate adaptive immune responses such as Th1 polarization and interferon gamma (IFNγ) production in CD4+ T cells, which further impairs insulin sensitivity (14, 15). Pro-inflammatory ATMs dominate obese adipose tissue and therefore therapeutic strategies that reduce or skew ATMs from M1 to anti-inflammatory M2 phenotypes have been reported to improve tissue homeostasis and metabolism (16, 17). Interestingly, work done by our group has shown that treatment of DIO mice with the CB1 antagonist SR141716A reverses obesity and is associated with a decrease in ATM-dependent inflammation in epididymal fat of high-fat diet (HFD)-induced obese mice (18).

Macrophages are heterogeneous myeloid-derived innate immune cells that are capable of polarizing between a broad spectrum of pro- (M1) and anti- (M2) inflammatory phenotypes. Dynamic switching of their polarization state and gene expression can be achieved through microRNA (miRNA, miR)- mediated regulation (19). miRNAs are short, non-coding RNAs that repress mRNA translation through complementary binding of a seed sequence with the 3′ untranslated region (3′UTR) of target mRNAs (20). Recently, we characterized dysregulated miRNAs in ATMs during DIO, which indicated miRNA expression in ATMs modulates their inflammatory phenotype (21). Importantly, miRNA-based therapeutics are currently being developed and tested for treatment of a variety of disorders including cardiovascular and metabolic diseases (22). Thus, identification of miRNA involved in DIO, may help develop miRNA-based treatment modalities.

Notch signaling is an evolutionarily conserved signaling pathway that is involved in cellular development and cell-cell communication. It regulates a variety of cellular processes such as proliferation, differentiation, survival, and inflammation (23). The Notch ligand Delta-like 4 (DLL4) contributes to many cardiometabolic disease mechanisms. In macrophages, DLL4 expression can be induced by M1-promoting stimuli such as the toll-like receptor 4 (TLR4) ligands lipopolysaccharide (LPS), IL-1β, and minimally modified low-density lipoprotein (24). Macrophage DLL4-Notch signaling promotes a feed-forward loop of inflammatory polarization through nuclear factor κB and interferon regulatory factor 8 transcriptional pathways leading to production of pro-inflammatory cytokines such as TNFα and CCL2 [(24, 25), p. 8]. Additionally DLL4+ macrophages can signal through Notch1 on other cell types such as adipocytes and CD4+ T cells to promote excess energy storage and Th1 polarization, respectively (14, 26–28). Thus, targeting the DLL4-Notch1 signaling pathway would be highly beneficial in treatment of cardiometabolic disorders. DLL4 blockade has been shown to improve atherosclerosis and metabolic disease while Notch1 blockade promotes browning of white adipose tissue and improves energy expenditure and metabolism (26, 29). Interestingly, miRNAs that target DLL4 have also been identified and include the miR-30 family [(30), p. 4; (31), p. 4].

In the current study, we used a mouse model of DIO intervention to characterize miRNAs regulated by CB1 blockade in ATMs. DIO mice were treated with the high affinity CB1 antagonist AM251. We found several miRNAs to be differentially expressed following AM251 treatment that were independent of food restriction. Notably, miR-30e-5p was induced following AM251 treatment in DIO mice and was accompanied by a decrease in pro-inflammatory gene expression and reduced plasma IFNγ concentration. CB1 blockade in macrophages also suppressed Dll4-mediated Th1 polarization in lymphocytes. Our findings support a role for CB1 in regulation of miRNAs that dampen ATM inflammation and improve metabolism, which suggests that manipulation of miRNA in ATMs could be used to treat DIO and other inflammatory disorders.

## Materials and Methods

### Mice

Sixteen-week-old C57Bl/6J DIO (JAX Stock # 380050 & 380056) mice were obtained from The Jackson Laboratory and maintained on purified diets of either HFD (60% kcal from fat, D12492, Research Diets) or LFD (10% kcal fat, D12450J, Research Diets). Mice were housed in an AAALAC-accredited, specific-pathogen-free facility at the University of South Carolina School of Medicine. After 2 weeks acclimation and 12-weeks of diet feeding, mice were treated with 10 mg/kg AM251 (1117, Tocris) or Vehicle by oral gavage daily for 4 weeks. At the conclusion of the study, mice were euthanized by overdose isoflurane inhalation. All experiments were performed according to protocols approved by the University of South Carolina Institutional Animal Care and Use Committee.

### Analytical Procedures

Body composition was determined by dual-energy x-ray absorptiometry (DEXA) in isoflurane-anesthetized mice. Body weight measurements were determined using an electronic gram scale with precision ±0.1 g. Blood glucose concentration was determined in 5 h fasted mice by applying ~5 μL tail-tip blood to a glucose test strip in a glucometer (Contour Next, Bayer). Fasting plasma insulin concentration was determined by ELISA kit according to manufacturer protocol including a standard curve (ThermoFisher Scientific). The homeostatic model assessment of insulin resistance (HOMA-IR) index was calculated by the equation (Fasting glucose x fasting insulin /405) (32).

### Adipose Tissue Dissociation

Stromal vascular fractions (SVF) of epididymal fat were isolated by collagenase digestion as previously detailed (21). In brief, minced adipose tissue was placed in Hank's Balanced Salt Solution containing 2% bovine serum albumin (BSA) and 1 mg/mL collagenase then homogenized with a gentleMACs dissociator (Miltenyi Biotec), and incubated for 30–40 min at 37°C with gentle shaking until fully dissociated. SVF cells were pelleted and floating adipocytes removed. SVFs were filtered, RBC-lysed, and washed, then used immediately for either flow cytometry or ATM isolation. To isolate ATMs, SVF cells were labeled with either Fluorescein isothiocyanate (FITC)- or Phycoerythrin (PE)-anti-F4/80 antibody (Clone: BM8, Biolegend) then F4/80+ ATMs were immunomagnetically selected with either FITC- or PE-Positive Selection Kit (StemCell Tech). Flow cytometry was used to evaluate selection purity, which was routinely >75%.

### Flow Cytometry

SVF cells or co-cultured cells were washed in staining buffer consisting of phosphate buffered saline (PBS), 2% heat-inactivated fetal bovine serum (FBS), and 1 mM EDTA. Blocking of Fc receptors was performed by incubation with TruStain FcX (BioLegend) for 10 m. Next, cells were incubated with appropriate fluorochrome-conjugated antibodies for 30 m on ice (CD45-APC/Cy7, clone: 30-F11; CD11b-AlexaFluor700, clone: M1/70; F4/80-BV421, clone BM8; T-bet-BV605, clone 4B10, CD4-APC-Cy7, clone GK1.5). Cells were washed 3X in staining buffer then analyzed on a BD FACSCelesta flow cytometer. Data was analyzed with FlowJo v10 software.

### RNA Purification, cDNA Synthesis, and qRT-PCR

ATMs were lysed in Qiazol and total RNA was purified with Qiagen miRNeasy Micro/Mini kit. RNA concentration and purity was measured using a NanoDrop 2000 spectrophotometer or Agilent Bioanalyzer. RNA was reverse transcribed to cDNA with the miScript II RT kit (Qiagen). Quantitative RT-PCR was performed with miScript SYBR Green PCR kit (Qiagen) or SSO Advanced Universal SYBR Green (BioRad). MiScript miRNA Primer Assays (Qiagen) were used for miRNA qRT-PCR. Genes were amplified with the following primers purchased from Integrated DNA Technologies—Beta-Actin: Fwd-GGCTGTATTCCCCTCCATCG, Rev-CCAGTTGGTAACAATGCCATGT; *Tnfa*: Fwd-CTGAACTTCGGGGTGATCGG, Rev-GGCTTGTCACTCGAATTTTGAGA; *IL6*: Fwd-CCAAGAGGTGAGTGCTTCCC, Rev-CTGTTGTTCAGACTCTCTCCCT; *Ccl2*: Fwd-TTAAAAACCTGGATCGGAACCAA, Rev-GCATTAGCTTCAGATTTACGGGT; *Ccl3*: Fwd-TTCTCTGTACCATGACACTCTGC, Rev-CGTGGAATCTTCCGGCTGTAG. Fold change in expression was determined by the comparative cycle method (2^−ΔΔ*Ct*^).

### MicroRNA Microarrays and Analysis

ATM RNA was prepared for microRNA microarrays and microarrays performed as previously described (18). Probe signal values were used to determine linear fold change between pairwise comparisons. Log_2_ fold change values were then calculated. MiRNAs were considered differentially expressed between groups if the Log_2_ fold change was at least ±2. Mean normalized expression (MNE) was calculated for each of the dysregulated miRs and a heatmap displaying MNE was made with GraphPad Prism Version 7.000 (GraphPad Software).

### Immunofluorescence and Histology

Epididymal fat pads were fixed in 4% paraformaldehyde and embedded in paraffin. Five micrometer tissue sections were cut, deparaffinized, and rehydrated then incubated in blocking buffer consisting of 1X PBS and 1% (w/v) BSA for 30 m. Tissues were then incubated with 1 μg TruStain FcX (Clone: 93, BioLegend) for 10 m followed by primary antibodies (F4/80-AlexaFluor488, clone: BM8, [1:100], BioLegend; DLL4-unconjugated, clone: HMD4-1, [1:100] BioLegend) for 30 m at room temperature. DLL4 signal was amplified with Biotin Goat-anti-Armenian Hamster IgG (Clone: Poly4055, [1:500], BioLegend) followed by Streptavidin-DyLight 633 [1:1,000], Invitrogen 21844). Tissues were counterstained with 40 μM Hoechst 33342 (Molecular Probes H21492), and 2 μg Phalloidin-tetramethylrhodamine (Sigma P1951), then mounted with ProLong Diamond Anti-fade Mountant (ThermoFisher P36965). Hematoxylin and eosin (H&E) staining of epididymal adipose tissue sections was performed by the University of South Carolina Instrumentation Resource Facility under standard protocols. Adipocyte size was quantified using the Adiposoft plugin in FIJI (FIJI is Just ImageJ, NIH) (33).

### Confocal Imaging and Analysis

Confocal microscopy and analysis were performed as previously described (21). In brief, confocal images of sectioned epididymal adipose tissue were taken on a Zeiss LSM 510 Meta Confocal Scanning Laser Microscope. Five images per sample were acquired using a 40X water immersion objective. Original LSM data files were imported into FIJI then split into channels. Thresholds were applied to the Cy5 channel using FIJI's max Entropy algorithm to identify regions of interest (ROI) that express DLL4. Then, area was measured for each ROI. Each biological replicate is the mean expression of five images.

### Macrophage: Lymphocyte Co-culture

Bone marrow derived macrophages (BMDM) were generated by isolating bone marrow cells from the tibia and femur of 6–8 week old naïve female C57Bl6/J mice and culturing in complete DMEM/F12 medium supplemented with 10% heat-inactivated FBS, 1% penicillin/streptomycin, 2 mM L-glutamine, and 1U/mL macrophage-colony stimulating factor (M-CSF, Biolegend 576406) for 7 days as previously described (21). Lymphocytes from mesenteric, inguinal, axillary, and cervical lymph nodes were isolated from naïve 6–8 week old female C57Bl6/J mice as previously described (34). Day 7 BMDM were re-plated in complete RPMI 1640 medium supplemented with 10% heat-inactivated FBS, 1% penicillin/streptomycin, 2 mM L-glutamine, 10 mM HEPES buffer, and 0.0002% β-mercaptoethanol. Next the BMDM were pretreated with AM251 (10 μM), α-DLL4 blocking mAb (1 μg/mL), AM251 (10 μM) + α-DLL4 (1 μg/mL), or appropriate vehicle and isotype controls for 1 hr. Then, polarization factors were added to the medium. For polarization to M1, 100 ng/mL LPS+ 50 ng/mL IFNγ was added (eBioscience 00-4976 and BioLegend 575302). For M2, 10 ng/mL IL-4 was added (BioLegend 574302). The cells were cultured for 24 h then BMDM supernatant was collected and the BMDM were washed, counted, and co-cultured at ratios 1:1 or 1:3 (BMDM:lymphocyte) with naïve lymphocytes. 1 × 10^5^ macrophages were cultured with either 1 × 10^5^ lymphocytes (1:1) or 3 × 10^5^ lymphocytes (1:3). ConcanavalinA (ConA, Sigma C5275) was added to the culture at a dose of 1 μg/mL to induce Th1 cell polarization. After 48 h of co-culture, the cells were collected for flow cytometry and supernatants were isolated for IFNγ ELISA (BioLegend 430801). BMDM supernatants were used for TNFα ELISA to confirm M1 polarization.

### Statistical Analysis

Statistical analyses were performed using GraphPad Prism Version 7.000 for Mac (GraphPad Software). Values are expressed as mean ± standard error. One-way ANOVA was used for multiple group analyses. Two-way ANOVA was used for significance across time points. The null hypothesis was rejected if *p* < 0.05.

## Results

### CB1 Blockade With AM251 Reverses DIO

**Figure 1:**
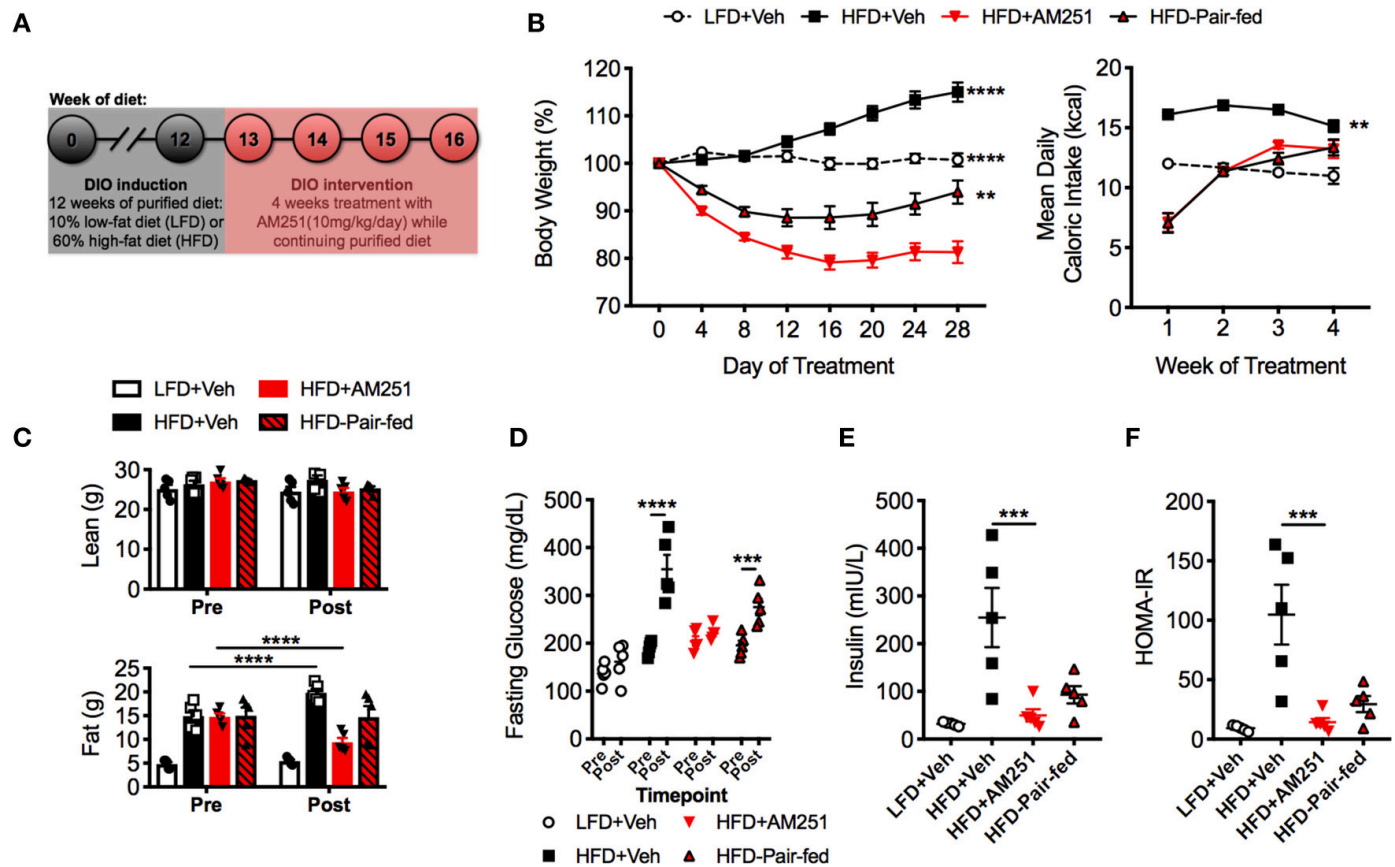
Treatment of mice with the CB1 antagonist (AM251) reverses obesity. 12-week HFD-fed obese mice were treated with the CB1 antagonist AM251 (10 mg/kg) for 4 weeks to assess intervention of obesity parameters. **(A)** Experimental timeline. **(B)** Percentage body weight growth and energy intake during the 4-week intervention. **(C)** DEXA body composition Pre- and Post- 4 weeks of treatment. **(D)** 5 h fasting blood glucose Pre- and Post- 4 weeks of treatment. **(E)** 5 h fasting insulin after 4 weeks of treatment. **(F)** Insulin resistance index (HOMA-IR) after 4 weeks of treatment. Data are mean ± SEM. *N* = 5 mice/group. Significance vs. HFD + AM251 was determined by one-way ANOVA **(B,E,F)**. Significance between time points was determined two-way ANOVA **(C,D)**. ^****^P < 0.0001, ^***^P < 0.001, ^**^P < 0.01.

To study the anti-obesity and anti-inflammatory effects of weight loss due to CB1 blockade, we used a well-established DIO intervention model whereby 6-week-old mice were fed HFD or control LFD for 12 weeks to induce an obese or lean phenotype, respectively. Following 12 weeks of purified diet feeding, baseline body composition was measured by DEXA and HFD-fed mice were stratified into experimental groups with equivalent mean fat mass. The mice were then treated with the CB1 antagonist AM251 at a dose of 10 mg/kg or Vehicle (Veh, 0.1% Tween80) for 4-weeks by daily oral gavage while continuing purified diet (Figure 1A). The experimental groups included: ad libitium fed LFD + Veh (lean reference), *ad libitum* fed HFD + Veh, *ad libitum* fed HFD + AM251, and a Vehicle-treated HFD group that was pair-fed to the HFD + AM251 group (called “HFD-Pair-fed”).

During the study, LFD + Veh mice maintained their initial body weight and HFD + Veh mice gained 15% of their baseline weight (Figure 1B). In contrast, HFD + AM251 mice experienced rapid and sustained weight loss during the 4-week DIO intervention model (Figure 1B). Acute weight loss was observed during the first week, which coincided with intense suppression of appetite (Figures 1B,C). For the remainder of the study, AM251-treated mice experienced persistent weight loss of ~20% their starting weight despite an increase in appetite (Figure 1B). Furthermore, appetite-independent weight loss was observed in HFD + AM251 mice as the HFD-Pair-fed group lost significantly less weight than the AM251-treated mice (Figure 1B). At the end of the study, the HFD-Pair-fed group was ~94% of it's baseline weight. Body weight changes were consistent with alterations in adiposity and not lean mass (Figure 1C). DEXA scans showed that fat mass was increased in HFD + Veh mice, decreased in HFD + AM251 mice, and unchanged in LFD + Veh and HFD-Pair-fed mice when compared to baseline measures (Figure 1C).

Glucose intolerance and decreased insulin sensitivity are hallmarks of the obese state. To evaluate AM251-dependent effects on glucose metabolism; fasting glucose, fasting insulin, and a homeostatic model assessment of insulin resistance index (HOMA-IR) were determined. HFD + AM251 mice did not experience an increase in fasting glucose over the duration of the 4-week intervention, whereas HFD + Veh and HFD-Pair-fed experienced significant increases in fasting glucose (Figure 1D). AM251-treatment in HFD-fed mice also led to decreases in fasting insulin and HOMA-IR measurements that were similar to LFD + Veh mice (Figures 1E,F). Although, these decreases may be primarily an effect of decreased appetite as HFD + AM251 measures were similar to those in HFD-Pair-fed mice (Figures 1E,F).

### AM251 Treatment in DIO Mice Reduces Inflammatory Adipose Tissue Macrophage Accumulation

**Figure 2:**
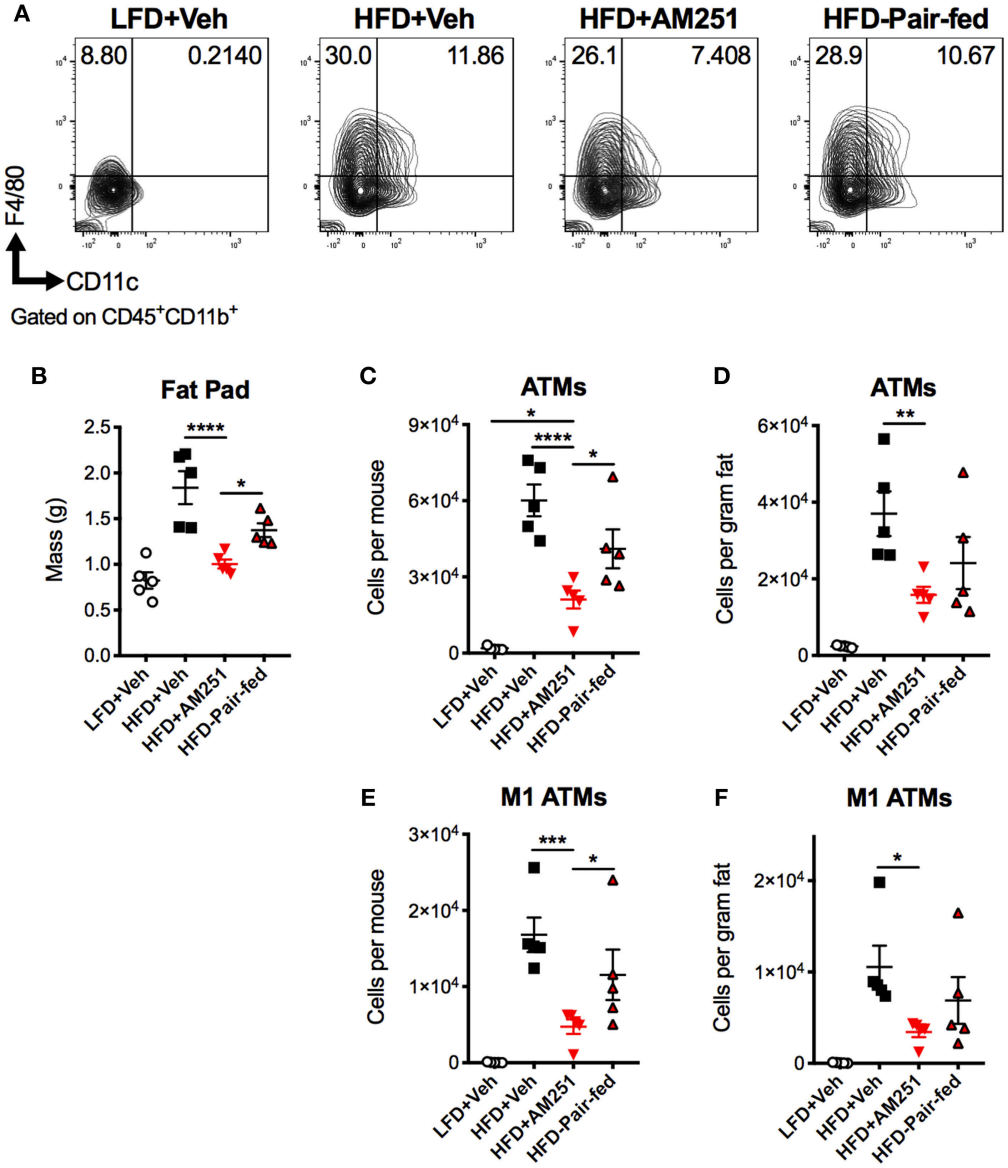
CB1 blockade reduces abundance of pro-inflammatory macrophages in epididymal fat. HFD-fed obese mice were treated with the CB1 antagonist AM251 (10mg/kg) as described in Figure 1 legend. Epididymal fat SVFs from AM251-treated DIO mice were analyzed by flow cytometry. CD45+CD11b+F4/80+ cells are considered as “ATMs”. CD45+CD11b+F4/80+CD11c+ cells are depicted as “M1 ATMs.” **(A)** Contour plots of ATMs. **(B)** Epididymal fat mass. **(C)** Total epididymal fat ATMs per mouse. **(D)** ATMs per gram of fat. **(E)** Total epididymal fat M1 ATMs per mouse. **(F)** M1 ATMs per gram of fat. Data are mean ± SEM. *N* = 5 mice/group. Significance was determined by one-way ANOVA. ^****^P < 0.0001, ^***^P < 0.001, ^**^P < 0.01, ^*^P < 0.05.

A chief characteristic of obesity is adipose tissue inflammation, which can lead to metabolic dysfunction. ATMs are the key initiators and instigators of this inflammation as they typically dominate obese adipose tissue through monocyte recruitment and local proliferation (12, 13). To determine macrophage burden in adipose tissue, SVF of epididymal fat were isolated after the 4-week intervention and subjected to flow cytometry analysis (Figure 2A). The percentages and total cell numbers of CD45^+^CD11b^+^F4/80^+^ ATMs and CD45^+^CD11b^+^F4/80^+^CD11c^+^ M1 ATMs in SVFs were elevated in HFD + Veh mice vs. LFD + Veh mice, and reduced in HFD + AM251 mice vs. HFD + Veh and HFD-Pair-fed (Figures 2A,C,E). We did not observe significant changes in CD45^+^CD11b^+^F4/80^+^CD11c^−^CD206^+^ M2 macrophages (data not shown). Epididymal fat pad mass was also reduced in AM251-treated mice (Figure 2B). To account for alterations in fat mass, ATM number per gram of adipose tissue was calculated and likewise AM251 treatment led to a reduction in ATM and M1 ATM numbers that were significant when compared to HFD+Veh group and trended less than HFD-Pair-fed group (Figures 2D,F).

### AM251 Intervention Modulates miRNA Expression in ATMs

Next we determined if AM251-intervention alters miRNA expression in ATMs as we have previously shown that miRNA dysregulation is observed in ATMs from obese mice and contributes to their inflammatory state (21). To perform this, F4/80+ ATMs were isolated from epididymal fat and used for miRNA microarray analysis. Of ~3,000 miRs tested, 41 and 25 were found to be up- and down-regulated, respectively in HFD + AM251 vs. HFD + Veh. Additionally, 123 and 42 were up- and down-regulated, respectively, in HFD + AM251 vs. HFD-Pair-fed. Also, 196 were up- and 70 were down-regulated in LFD + Veh vs. HFD + Veh.

**Figure 3:**
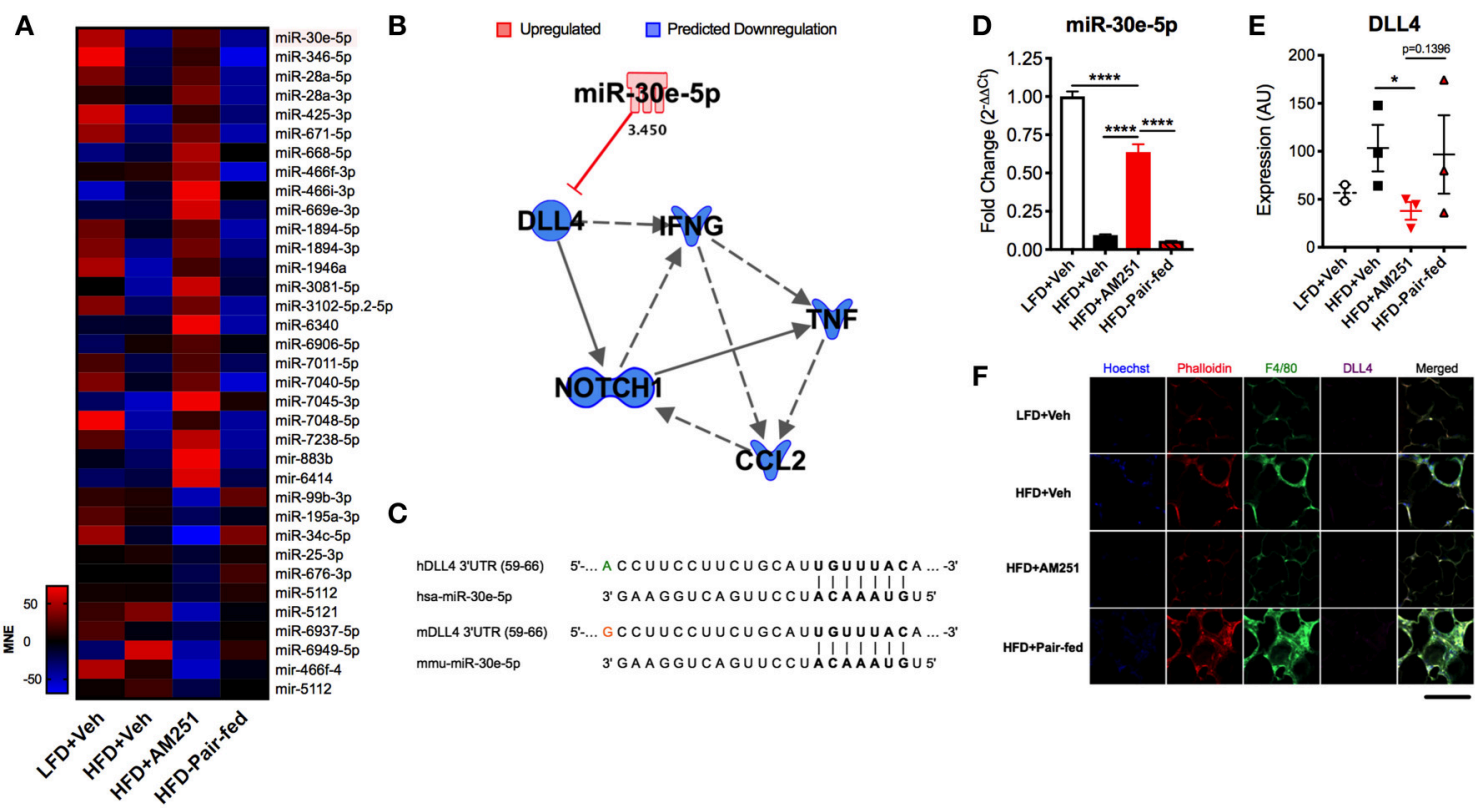
AM251 treatment elevates ATM miR-30e-5p and reduces target expression of the pro-inflammatory Notch ligand Delta-like-4. DIO mice were treated with the CB1 antagonist AM251 (10 mg/kg) as described in Figure 1 legend. MicroRNA microarrays were performed on F4/80+ ATMs isolated from epididymal fat after the 4-week AM251 intervention. **(A)** Heatmap of differentially expressed miRs in ATMs resulting from AM251 treatment, independent of effects of caloric restriction (Log_2_ fold change ≥±2). **(B)** Qiagen IPA network of miRs targeting the DLL4-Notch signaling pathway and downstream inflammatory cytokines. Expression values and predictions are overlaid on the network. **(C)** Sequence conservation of human and mouse miR-30e-5p binding to Dll4 3′UTR. **(D)** qRT-PCR of miR-30e-5p in ATMs. **(E)** Confocal microscopy quantitation of DLL4 expression in epididymal adipose tissue. **(F)** Representative confocal micrographs. Images are to equal scale. For **(A,D)**, data are presented from independent experiments of 4–10 pooled mice per group. For **(E)**, *N* = 3 mice/group. Significance was determined by one-way ANOVA. ^****^P < 0.0001, ^*^P < 0.05. See also Supplementary Table 1 and Supplementary Figure 1.

To distinguish differentially expressed miRs in HFD + AM251 ATMs that were independent of appetite restriction, we identified miRs that were similarly dysregulated in HFD+AM251 vs. HFD + Veh and in HFD + AM251 vs. HFD-Pair-fed. This analysis led to identification of 35 miRNAs that were differentially expressed due to appetite-independent AM251 treatment. Of these 35 miRNAs, 24 were up-regulated and 11 were down-regulated (Figure 3A and Supplementary Table 1). A core analysis of these miRs with Qiagen Ingenuity Pathway Analysis (IPA, Qiagen) revealed significant overlap (*p* < 0.05) with processes such as cancer, inflammatory response, metabolic disease, and hepatitis (Supplementary Figures 1A,B). Furthermore, the top affected networks involved carbohydrate metabolism and cell cycle (Supplementary Figures 1C,D). Interestingly, miR-30e-5p expression was found to be elevated in HFD+AM251 (Figure 3A), which as per the IPA analysis was associated with DLL4-Notch signaling-induced inflammation (Figure 3B). In addition, miR-30 was found to target Dll4 3′UTR which was conserved among humans and mice (Figure 3C).

### AM251 Treatment Leads to Induction of miR-30e-5p and Reduces DLL4

Next, we validated expression of miR-30e-5p in F4/80+ ATMs by qRT-PCR (Figure 3D). Consequently, we evaluated protein expression of the miR-30e-5p target DLL4 in adipose tissue by immunofluorescence (Figures 3E,F). DLL4 expression was significantly decreased in HFD + AM251 vs. HFD + Veh (Figures 3E,F).

### CB1 Blockade Reduces Energy Storage and Type 1 Adipose Tissue Inflammation

**Figure 4:**
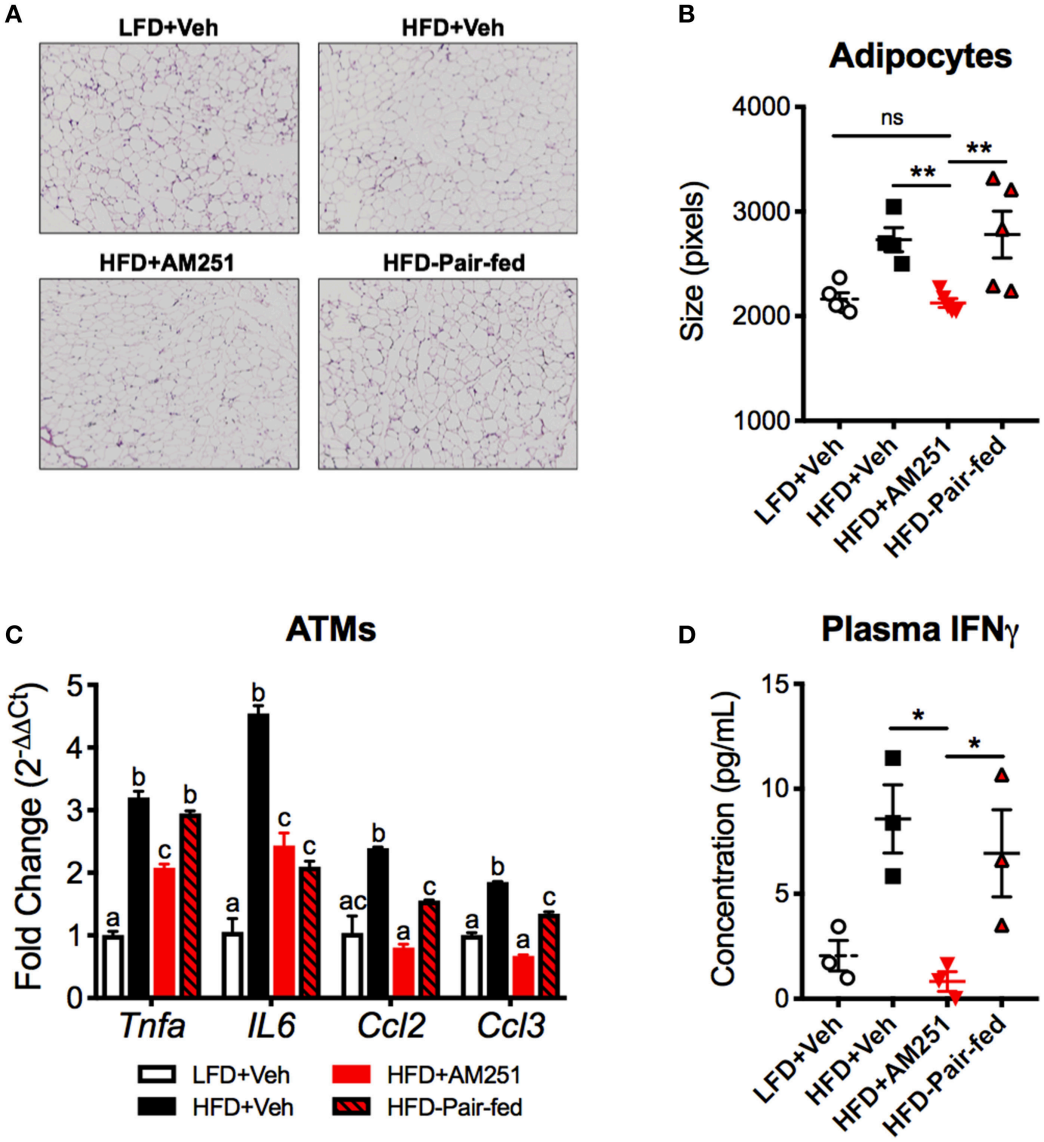
AM251 treatment *in vivo* reduces energy storage and type 1 inflammation associated with DLL4-Notch signaling. HFD-fed obese mice were treated with AM251 (10 mg/kg) for 4 weeks as described in Figure 1 legend. Adipocyte size, ATM gene expression, and plasma IFNγ were quantified in mice after the 4-week AM251 intervention. **(A)** Representative H & E stains of epididymal fat. Images are to equal scale (4X). **(B)** FIJI adipocyte size quantification. **(C)** qRT-PCR expression of inflammatory cytokine/chemokine genes in F4/80 + ATMs. **(D)** Plasma IFNγ concentration measured by ELISA. Data are mean ± SEM. For **(A,B)**, *N* = 4–5 mice/group. For **(C)**, data are presented from a single experiment of 4–10 pooled mice per group. For **(D)**, *N* = 3 independent experiments of pooled plasma from 4 to 10 mice/group. Significance was determined by one-way ANOVA. ^**^P < 0.01, ^*^P < 0.05. For **(C)**, *p* < 0.05 if alphabetical characters differ between groups.

Dll4-Notch1 signaling has been characterized to promote energy storage and type 1 inflammatory responses. Consistent with a decrease in DLL4, epididymal adipocyte size and fat mass was reduced following AM251 treatment (Figures 2B, 4A,B). We performed qRT-PCR of pro-inflammatory genes in ATMs, which revealed that *Tnfa, Ccl2, IL6*, and *CCL3* expression are reduced following AM251 treatment *in vivo* (Figure 4C). In accordance with a decrease in M1 ATM activation and DLL4 expression, the Th1 response was reduced in HFD + AM251 vs. HFD + Veh-treated mice as evidenced by decreased levels of circulating IFNγ (Figure 4D).

### AM251 Reduces Th1 Inflammation by Regulating Macrophage DLL4

**Figure 5:**
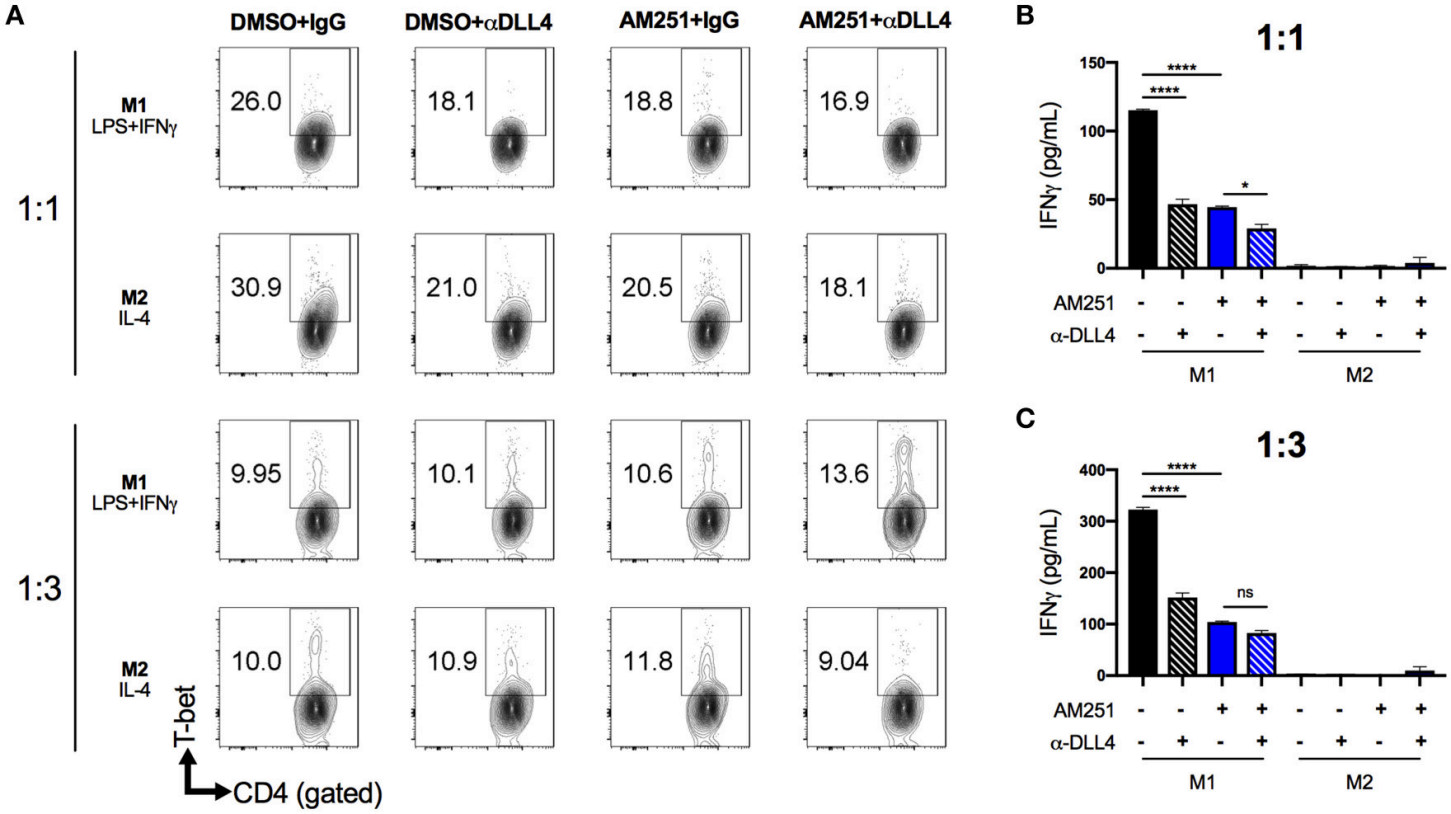
AM251 reduces expansion of pro-inflammatory Th1 cells by downregulating DLL4 in macrophages. BMDM from naïve mice were pretreated with AM251 (10 μM) ± α-DLL4 blocking mAb (1 μg/mL) or vehicle/isotype controls for 1 h then polarized to M1 (LPS + IFNγ) or M2 (IL-4) for 24 h. The polarization medium was then washed away and BMDM were co-cultured with naïve lymphocytes at ratios of 1:1 or 1:3 (BMDM:lymphocytes) in the presence of 1 μg/mL ConA for 48 hr. Resulting Th1 polarization of CD4+ lymphocytes was assessed. **(A)** Flow cytometry of CD4+T-bet+ Th1 lymphocytes. **(B,C)** IFNγ concentration in cell supernatants. Data are mean ± SEM. ^****^p < 0.0001, ^*^p < 0.05 by one-way ANOVA. See also Supplementary Figure 3 for lymphocyte ConA controls.

To test the possibility that AM251-mediated suppression of DLL4 was causing reduced Th1 polarization, we pre-treated naïve BMDM with anti-DLL4 blocking antibody (α-DLL4), AM251, or AM251 + α-DLL4, then added polarization factors to the medium to promote M1 (LPS + IFNγ) or M2 (IL-4) phenotypes. After 24 h of polarization, the BMDM supernatant was collected and used for TNFα ELISA to confirm M1 polarization (Supplementary Figure 2). Next, the BMDMs were washed and co-cultured with naïve lymphocytes in the presence of the T-cell mitogen, ConA for 48 h. Culture of lymphocytes alone with ConA was able to induce a Th1 response as evidenced by elevated T-bet expression and IFNγ secretion (Supplementary Figure 3). The most Th1 polarization was observed in the M1-DMSO+IgG vehicle/Isotype control co-culture (Figures 5A–C). Blocking DLL4 signaling (M1-DMSO+α-DLL4) caused reduced Th1 polarization (Figures 5A–C). Similarly, CB1 blockade with AM251 reduced Th1 polarization to the same extent as blocking DLL4 alone (Figures 5A–C). Blocking of both CB1 and DLL4 led to little change in Th1 polarization vs. blocking of CB1 alone or DLL4 alone (Figures 5A–C). As expected, lymphocyte IFNγ production was observed only in M1 co-cultures as M2 co-cultures served as a negative control (Figures 5B,C).

## Discussion

ATMs are a dominant cell type in the adipose tissue of obese individuals and their production of inflammatory mediators contributes to metabolic dysfunction and progression toward development of cardiometabolic disorders. Therapies that decrease ATM-dependent inflammation are thought to have great potential for decreasing the incidence of obesity and complications (35). In the current study, we identified miRNAs in ATMs that are associated with decreased inflammation and weight-loss due to pharmacological intervention of DIO with the CB1 antagonist AM251.

The CB1 receptor is well-known to modulate appetite and energy homeostasis. Blockade of CB1 activation attenuates obesity by affecting multiple areas including leptin signaling, white adipose tissue browning, gut microbiota interactions, and reducing inflammation (5, 6, 8, 9, 18, 36). In this study and consistent with previous reports, we found that AM251 induces weight loss, improves glucose metabolism, and decreases adipose tissue inflammation.

To further understand regulation of ATM inflammation in this model, we investigated miRNAs by use of microarrays. MiRNAs are important regulators of gene expression because it is thought that ~60% of genes are conserved targets for miRNAs (20). Alterations in miRNA expression can also lead to quick changes in gene expression and use of miRNAs for therapeutic purposes holds high potential. We found that treatment of DIO mice with AM251 for 4-weeks led to various alterations of miRNA expression in ATMs from epididymal fat. Furthermore, use of a pair-fed control led to identification of several of miRs that were independent of AM251-induced appetite restriction. Therefore, we identified several miRNAs that may be involved in regulation of enhanced energy expenditure not due to calorie restriction. These miRNAs were also found to have overlap with several pathways relating to obesity-associated comorbid disorders including cancer, hematological disease, immunological disease, and metabolic diseases. Together these data indicate miRNA-mediated regulation of ATMs contribute to attenuation of obesity by reducing adipose tissue inflammation, improving energy metabolism, and inducing weight-loss during DIO intervention with AM251.

Excitingly, we found that miR-30e-5p was reproducibly induced following AM251 treatment, which indicates an association between miR-30e-5p upregulation and appetite-independent weight loss. This miRNA has been previously characterized to target DLL4 and reduce DLL4-Notch signaling (30, 31). We have also previously demonstrated that the miR-30 family is downregulated in ATMs from obese vs. lean mice, while DLL4 expression is increased in ATMs from obese vs. lean mice, which indicates that miR-30e-5p attenuates inflammation in ATMs through regulation of the Notch signaling pathway (21). Downregulation of miR-30 in macrophages also induced secretion of pro-inflammatory mediators TNFα and CCL2 (21). Here our results support our previous findings and excitingly, induction of miR-30e-5p in HFD + AM251 ATMs indicates that miR-30e-5p may be used therapeutically to dampen ATM-mediated inflammation and reduce obesity. Consistent with increased miR-30e-5p expression, we found that DLL4 expression was reduced in adipose tissue from HFD + AM251 mice. It is possible that miR-30e-5p could have additional beneficial effects beyond targeting *Dll4*, however, we found several parameters to be reduced in AM251 treated mice that are consistent with a reduction in DLL4-Notch signaling. These include a reduction in adipocyte size and fat pad mass, which indicates reduction in energy storage and an increase in energy expenditure, a reduction in ATM pro-inflammatory cytokine gene expression and M1 polarization, and a reduction in Th1 inflammation with reduced circulating IFNγ.

**Figure 6:**
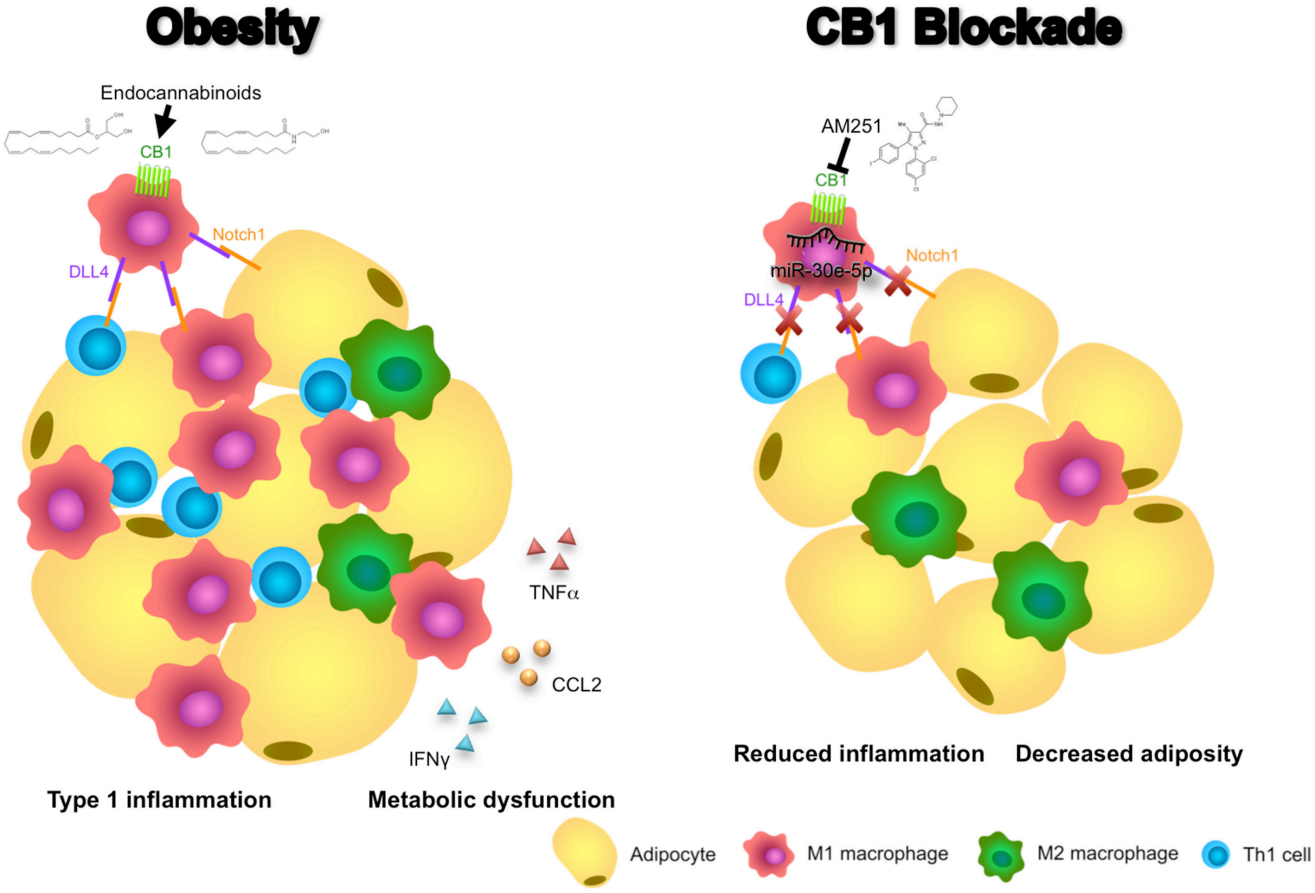
Blocking CB1 receptors reverses obesity through miR-30e-5p regulation of DLL4. In this illustrative summary we demonstrate that blocking of CB1 receptors can reverse the inflammation and metabolic dysfunction observed in obese adipose tissue. During obesity, endocannabinoids activate CB1 to promote pro-inflammatory M1 macrophages and upregulation of DLL4. Subsequently, DLL4 activates Notch signaling on other macrophages, T cells, and adipocytes to promote M1 polarization, Th1 polarization, and adipocyte hypertrophy, respectively. As a result, pro-inflammatory cytokines and chemokines such as TNFα, CCL2, and IFNγ, and fat mass expansion, lead to metabolic dysfunction. Conversely, blocking of CB1 with AM251 reverses DLL4-Notch signaling-mediated inflammation and metabolic impairments due to upregulation of miR-30e-5p and downregulation of DLL4. As a result, fewer M1 macrophages and Th1 cells reside in the adipose tissue and energy expenditure increases to reduce fat mass.

In addition, we demonstrated that blocking of CB1 receptors specifically on M1 macrophages could suppress their ability to promote a Th1 response in CD4+ T cells. This effect appears to be dependent on CB1-mediated regulation of DLL4 expression in macrophages as DLL4 is known to promote Th1 differentiation and we, in this study, showed that CB1 blockade reduces expression of this ligand (27). Thus, our data support a mechanism whereby CB1 blockade promotes induction of miR-30e-5p, which downregulates macrophage DLL4 and subsequent type 1 inflammatory responses including M1 macrophage and Th1 CD4+ T cell polarization, along with production of their inflammatory cytokines including TNFα, CCL2, and IFNγ (Figure 6).

Previous evidence advocates that miR-30 as well as DLL4 blockade may be therapeutically beneficial to reducing severity of metabolic disorders. MiRNA-30c mitigates hypercholesterolemia and atherosclerosis in mice by altering lipid metabolism (37, 38). Additionally, miR-30b/c promotes thermogenesis and adipose tissue browning (30, 39). Moreover, miR-30 expression in ATMs is inversely correlated with obesity (21). DLL4 blockade also protects against atherosclerosis (29). Blocking the DLL4 receptor Notch1 also attenuates obesity (26). Together, these data indicate that blocking of CB1 in ATMs may contribute to improvement in obesity phenotype through miR-30e-5p regulation of DLL4. Therefore, future studies focusing on *in vivo* induction of macrophage-specific miR-30 in DIO and other cardiometabolic models could shed new light on the mechanistic potential of this miRNA family for treatment of these macrophage-driven chronic diseases.

## Data Availability

The data discussed in this publication have been deposited in NCBI's Gene Expression Omnibus (40) and are accessible through GEO Series accession number GSE129273 (https://www.ncbi.nlm.nih.gov/geo/query/acc.cgi?acc=GSE129273).

## Ethics Statement

All experiments were performed according to protocols approved by the University of South Carolina Institutional Animal Care and Use Committee.

## Author Contributions

KM, PM-B, PN, and MN: conceptualization and methodology. KM: validation, formal analysis, writing- original draft, and visualization. KM and PM-B: investigation. PN and MN: resources, supervision, and funding acquisition. KM, PN, and MN: writing- review and editing.

### Conflict of Interest Statement

The authors declare that the research was conducted in the absence of any commercial or financial relationships that could be construed as a potential conflict of interest.
