# Supplementary material for: Cannabinoid Receptor 1 Blockade Attenuates Obesity and Adipose Tissue Type 1 Inflammation Through miR-30e-5p Regulation of Delta-Like-4 in Macrophages and Consequently Downregulation of Th1 Cells
Source: Front Immunol. 2019 May 10;10:1049. doi: 10.3389/fimmu.2019.01049 (PMC6523050; doi:10.3389/fimmu.2019.01049)
Supplement: Supplementary file 1 [file Data_Sheet_1.PDF]

Supplementary Material

1 Supplementary Table 1

Table S1.	Comparison (Log$_2$ fold change)		
MicroRNA ID	LFD+Veh vs. HFD+Veh	HFD+AM251 vs. HFD+Veh	HFD+AM251 vs. HFD-Pair-fed
mmu-miR-30e-5p	6.386	3.450	3.880
mmu-miR-346-5p	6.979	2.131	5.107
mmu-miR-28a-5p	3.288	2.652	4.274
mmu-miR-28a-3p	1.451	2.253	4.292
mmu-miR-425-3p	4.917	2.348	2.021
mmu-miR-671-5p	3.631	2.923	4.117
mmu-miR-668-5p	-1.409	3.236	2.456
mmu-miR-466f-3p	-1.112	2.674	22.225
mmu-miR-466i-3p	-2.023	4.668	3.614
mmu-miR-669e-3p	-1.026	2.142	2.390
mmu-miR-1894-5p	2.554	2.220	5.140
mmu-miR-1894-3p	4.814	4.405	4.637
mmu-miR-1946a	7.775	4.164	2.332
mmu-miR-3081-5p	1.690	3.287	2.353
mmu-miR-3102-5p.2-5p	4.134	3.661	5.222
mmu-miR-6340	-1.007	2.453	3.541
mmu-miR-6906-5p	-3.464	2.037	3.136
mmu-miR-7011-5p	2.712	2.793	3.109
mmu-miR-7040-5p	4.105	3.438	15.522
mmu-miR-7045-3p	1.856	17.438	4.285
mmu-miR-7048-5p	7.016	2.653	2.538
mmu-miR-7238-5p	2.811	4.674	5.337
mmu-mir-883b	1.243	3.196	3.699
mmu-mir-6414	-1.073	2.080	2.122
mmu-miR-99b-3p	1.082	-2.413	-3.258
mmu-miR-195a-3p	2.502	-4.601	-2.357
mmu-miR-34c-5p	5.540	-5.626	-23.750
mmu-miR-25-3p	-1.300	-2.325	-2.021
mmu-miR-676-3p	1.003	-2.051	-3.930
mmu-miR-5112	1.064	-2.500	-3.202
mmu-miR-5121	-1.968	-16.757	-4.637
mmu-miR-6937-5p	3.597	-2.075	-2.827
mmu-miR-6949-5p	-3.261	-4.095	-2.201
mmu-mir-466f-4	1.979	-2.734	-2.182
mmu-mir-5112	-1.837	-4.554	-2.165
Upregulated			
Downregulated			

Table S1. Dysregulated miRNAs and their experimental fold change observations in F4/80$^+$ ATMs.

2 Supplementary Figure 1

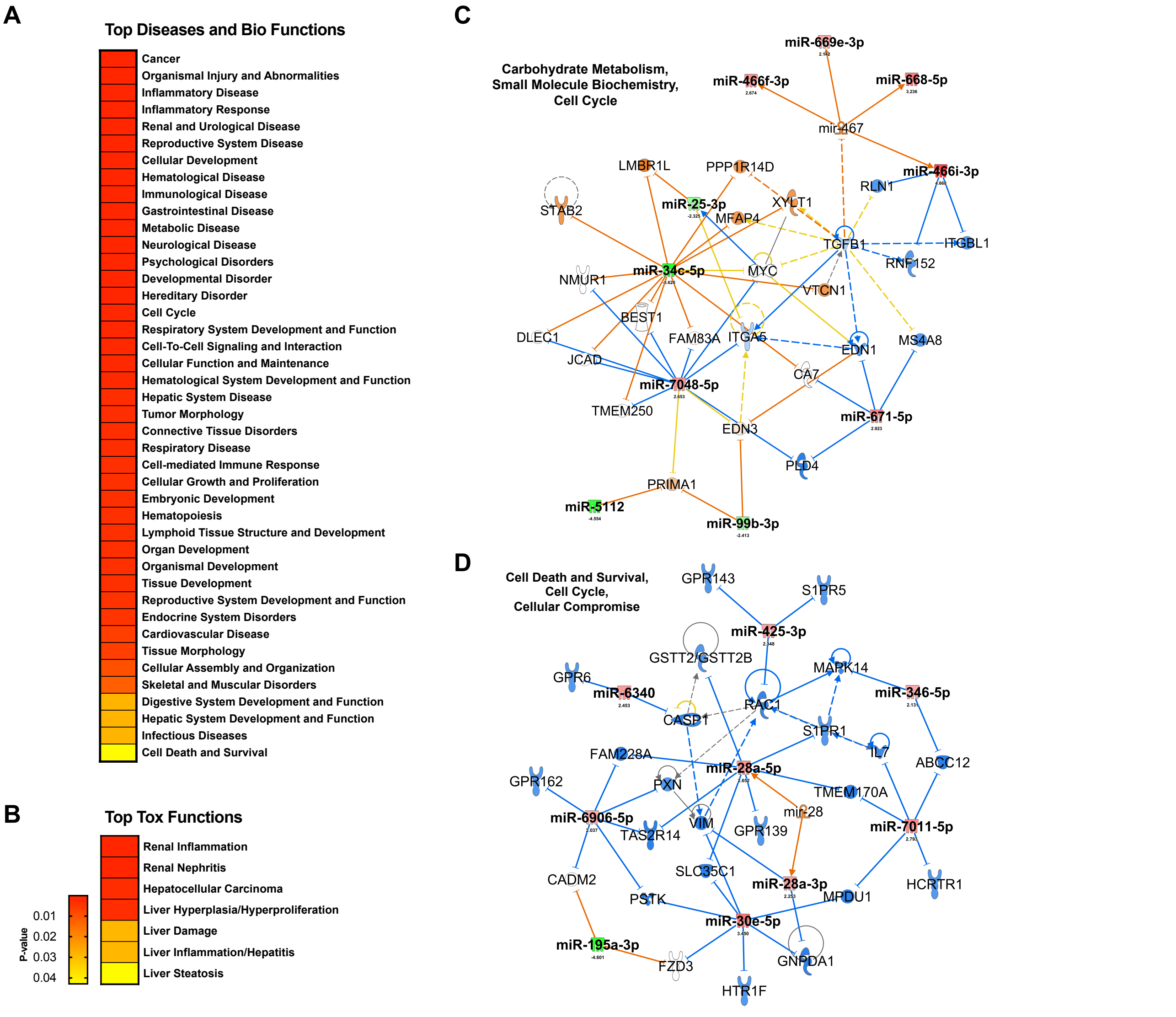

Figure S1. Pathway analysis of dysregulated miRNAs following AM251 treatment.

Qiagen IPA was used for core analysis of dysregulated miRNAs in ATMs. (A-B) Top Diseases, biological functions, and toxicological functions affected by the dysregulated miRs. (C-D) The top 2 affected networks. Network pathways are depicted with overlaid expression values and predictions. Red: upregulated, green: downregulated, orange: predicted upregulation, blue: predicted downregulation.

3 Supplementary Figure 2

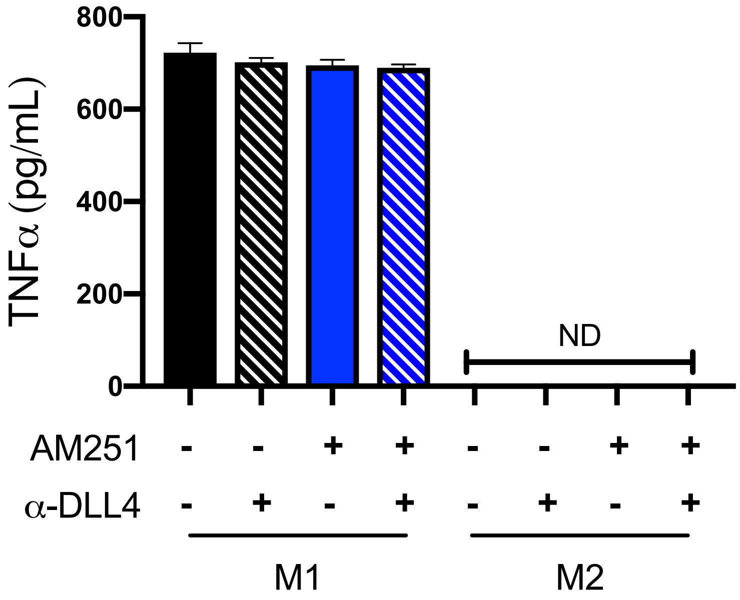

Figure S2. Validation of M1 polarization in BMDM.

BMDM from naïve mice were pretreated and polarized as described in Main Figure 5. BMDM supernatants were used for TNFα ELISA to confirm M1 polarization of macrophages.

4 Supplementary Figure 2

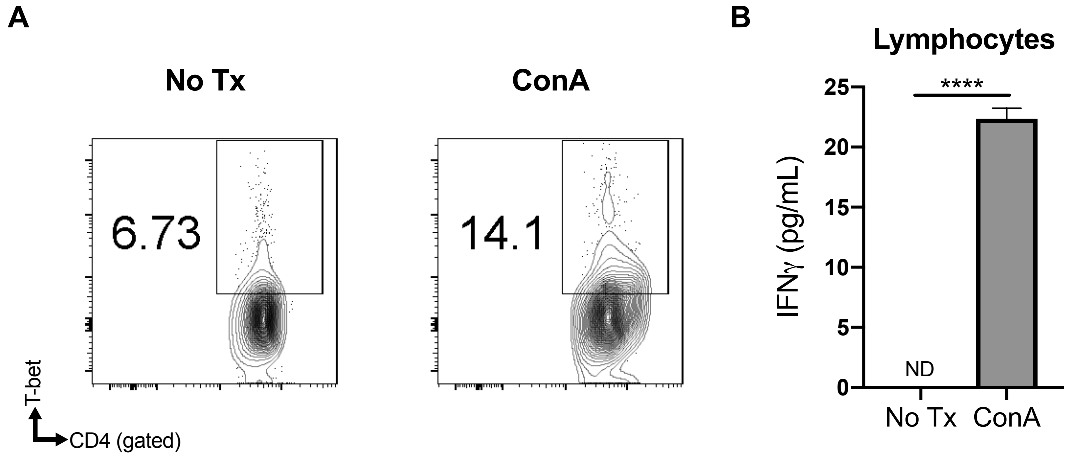

Figure S3. Concanavalin A is a positive control for induction of Th1 lymphocytes.

Naïve lymphocytes from Main Figure 5 were cultured in medium containing 1ug/mL ConA for 48 hr. (A) Flow cytometry of $CD4^{+}$T-bet^{+} Th1 lymphocytes. (B) IFNγ concentration detected in cell supernatant by ELISA. ****$p<0.0001$ by unpaired two-tailed T-test. ND: not detected.
